# Gonorrhea - an evolving disease of the new millennium

**DOI:** 10.15698/mic2016.09.524

**Published:** 2016-09-05

**Authors:** Stuart A. Hill, Thao L. Masters, Jenny Wachter

**Affiliations:** 1 Department of Epidemiology, Gillings School of Global Public Health, University of North Carolina at Chapel Hill, Chapel Hill, NC 27599-7435.

**Keywords:** pathogenesis, antigenic variation, immune manipulation, antibiotic resistance, panmictic

## Abstract

Etiology, transmission and protection: *Neisseria gonorrhoeae* (the gonococcus) is the etiological agent for the strictly human sexually transmitted disease gonorrhea. Infections lead to limited immunity, therefore individuals can become repeatedly infected. Pathology/symptomatology: Gonorrhea is generally a non-complicated mucosal infection with a pustular discharge. More severe sequellae include salpingitis and pelvic inflammatory disease which may lead to sterility and/or ectopic pregnancy. Occasionally, the organism can disseminate as a bloodstream infection. Epidemiology, incidence and prevalence: Gonorrhea is a global disease infecting approximately 60 million people annually. In the United States there are approximately 300, 000 cases each year, with an incidence of approximately 100 cases per 100,000 population. Treatment and curability: Gonorrhea is susceptible to an array of antibiotics. Antibiotic resistance is becoming a major problem and there are fears that the gonococcus will become the next “superbug” as the antibiotic arsenal diminishes. Currently, third generation extended-spectrum cephalosporins are being prescribed. Molecular mechanisms of infection: Gonococci elaborate numerous strategies to thwart the immune system. The organism engages in extensive phase (on/off switching) and antigenic variation of several surface antigens. The organism expresses IgA protease which cleaves mucosal antibody. The organism can become serum resistant due to its ability to sialylate lipooligosaccharide in conjunction with its ability to subvert complement activation. The gonococcus can survive within neutrophils as well as in several other lymphocytic cells. The organism manipulates the immune response such that no immune memory is generated which leads to a lack of protective immunity.

## INTRODUCTION

*Neisseria gonorrhoeae* (the gonococcus) is a Gram-negative diplococcus, an obligate human pathogen, and the etiologic agent of the sexually transmitted disease, gonorrhea. The gonococcus infects a diverse array of mucosal surfaces, some of which include the urethra, the endocervix, the pharynx, conjunctiva and the rectum 1. In 2013, the Centers for Disease Control and Prevention (CDC) reported that there were 333,004 new cases of gonorrhea in the United States, with an incidence of 106.1 cases per 100,000 population 2. Worldwide, 106.1 million people are infected by *N. gonorrhoeae* annually 3. In most cases, the disease is a noncomplicated mucosal infection. However, in a few patients, generally with women, more serious sequelae can occur and include salpingitis (acute inflammation of the fallopian tubes), pelvic inflammatory disease (PID; an infection in the upper part of the female reproductive system), or, in rare cases, as a bacteremic infection 4. If left untreated, these more serious complications can result in sterility, ectopic pregnancy, septic arthritis, and occasionally death. Approximately 3% of women presenting with a urogenital infection develop the most severe forms of the disease 5. However, the occurrence of PID has significantly decreased over time 6,7,8, with an estimated 40,000 cases of infertility in women annually 9. Dissemination rarely occurs, but when the bacteria do cross the endothelium, they can spread to other locations in the body. Currently, a more worrying trend has emerged, in that, there now appears to be an increased risk for HIV infection in patients that are also infected with *N. gonorrhoeae* 10.

Gonorrhea the disease was initially described approximately 3,500 years ago, but it was not until 1879 that Albert Neisser determined the etiologic agent of the disease 11. The *Neisseriae* are usually regarded as microaerophilic organisms. However, under the appropriate conditions, they are capable of anaerobic growth 12. *In vitro* cultivation of this fastidious organism has always been problematic and it was not until the development of an improved Thayer-Martin medium that early epidemiological studies could be undertaken. Subsequently, other commercial growth mediums have since been developed which has allowed for a greater understanding of the disease process.

## VIRULENCE FACTORS OF *N. GONORRHOEAE*

Like many Gram-negative bacterial pathogens, *N. gonorrhoeae* possesses a wide range of virulence determinants, which include the elaboration of pili, Opa protein expression, lipooligosaccharide expression (LOS), Por protein expression and IgA1 protease production that facilitates adaptation within the host.

### Type IV pili (Tfp)

Considerable attention was paid to pili stemming from the observations of Kellogg and coworkers 12,13 that virulent (T1, T2 organisms) and avirulent (T3, T4 organisms) strains could be differentiated on the basis of colony morphology following growth on solid medium. Subsequently, it was established that all freshly isolated gonococci possessed thin hair-like appendages (pili) which were predominantly composed of protein initially called pilin but subsequently renamed PilE 14. The elaboration of pili is a critical requirement for infection as this structure plays a primary role in attaching to human mucosal epithelial cells 15, fallopian tube mucosa 16,17, vaginal epithelial cells 16,18 as well as to human polymorphonuclear leukocytes (PMN’s; neutrophils) 19,20. Due to their prominent surface location, pili were initially thought to be an ideal vaccine candidate as pilus-specific antibodies were observed in genital secretions 18. However, two prominent vaccine trials failed, with evidence indicating that pilus protein(s) underwent antigenic variation 21.

Gonococcal pili are categorized as Type IV pili, as the PilE polypeptide is initially synthesized with a short (7 amino acid) N-terminal leader peptide, which is then endo-proteolytically cleaved 22. The mature PilE polypeptide is then assembled at the inner membrane into an emerging pilus organelle with the PilE polypeptides being stacked in an α-helical array 23. The PilE polypeptide consists of three functional domains based on sequence characteristics 24. The N-terminal domain is highly conserved and is strongly hydrophobic, with this region of the protein comprising the core of the pilus structure 23. The central part of the PilE monomer is partially conserved and structurally aligned as a β-pleated sheet. As the C-terminal domain is hydrophilic, this segment of the protein is exposed to the external environment 23 and undergoes antigenic variation which allows the bacteria to avoid recognition by the human host’s immune cells (reviewed 25,26).

Assembly of the pilus structure is complicated and involves other proteins besides PilE (e.g., the pilus tip-located adhesion, PilC) 27 as well as other minor pilus components PilD, PilF, PilG, PilT, PilP and PilQ 28. During pilus biogenesis, and prior to assembly, the leader peptide is removed from PilE by the PilD peptidase 23. The N-terminal domain then facilitates translocation across the cytoplasmic membrane allowing PilE subunits to be polymerized at the inner membrane 29,30. As the pilus structure is assembled, it is extruded to the exterior of the outer membrane using the PilQ pore forming complex 29,30,31. PilC is a minor protein located at the tip of pilus as well as being present at its base. The *pilC* gene exists as 2 homologous, but non-identical copies, *pilC1* and *pilC2* in most gonococcal strains, with only the *pilC2* gene being expressed in piliated *N. gonorrhoeae *MS11 strains 27. *pilC* expression is also subject to RecA-independent phase variation (on/off switching) due to frequent frameshift mutations occurring within homo-guanine tracts located within its signal peptide region 27. PilC participates in pilus biogenesis as well as in host cell adherence, as *pilC* mutants prevent the formation of pili by negatively affecting their assembly process, which leads to the bacteria being unable to adhere to human epithelial cells 32.

In addition to promoting attachment to host cells, type IV pili are also involved in bacterial twitching motility, biofilm formation, and DNA transformation 33. *N. gonorrhoeae* is naturally competent for transformation in that it can take up exogenously produced *Neisseria*-specific DNA containing a 10-bp uptake sequence (GCCGTCTGAA; DUS) 34. *pilE* mutations resulting in loss of pilus expression lead to transformation incompetence 28,35. The binding and uptake of exogenous DNAs by *N. gonorrhoeae* requires type-IV-pili-structurally-related components, including ComP protein 36,37. Despite sharing sequence similarity to PilE in the N-terminal domain, ComP was shown to be dispensable to Tfp biogenesis 36. Instead the bacteria were unable to take up extraneous DNA; subsequent overexpression of ComP increased sequence-specific DNA binding, suggesting that ComP functions in the DNA binding step of transformation 37. Recently, ComP has been shown to preferentially bind to DUS-containing DNAs via an electropositive stripe on its surface 38 with uptake of the DNA being facilitated by de-polymerization of the pilus structure through PilT hydrolytic activity 39. The coordinated physical retraction and elongation of pili can lead to "twitching", a form of motility that propels the cell along a surface. Retraction is facilitated by PilT activity (an ATPase), whereas PilF protein promotes pilus elongation at the inner membrane 39,40.

### Por protein

The outer membrane porin protein, Por, is the most abundant protein in the gonococcus accounting for approximately 60% of the total protein content 1. The molecular size of Por varies between strains, yet, within individual strains, it exists as only a single protein species 41. Por has been used as the basis for serological classification of gonococci 41 with nine distinct serovars being identified 42. Overall, there are two distinct structural classes (PorA and PorB) 42, with the PorA subgroup tending to be associated with the more complicated aspects of the disease, whereas the PorB subgroup is more likely to be involved with uncomplicated mucosal infections 43.

Porins allow the transport of ions and nutrients across the outer membrane and can also contribute to the survival of the bacteria in host cells 44. Moreover, gonococcal Por protein has been shown to translocate from the outer membrane into artificial black lipid membranes 45 as well as into epithelial cell membranes, following attachment of the bacteria 46. Por can also transfer into mitochondria of infected cells which leads to the formation of porin channels in the mitochondrial inner membrane, causing increased permeability 47. This causes the release of cytochrome c and other proteins, leading to apoptosis of infected cells 48. However, Por-induced apoptosis remains controversial. In direct contrast to events with the gonococcus, *Neisseria meningitidis* Por, which also interacts with mitochondria, apparently protects cells from undergoing apoptosis 49. Interestingly, mitochondrial porins and *Neisseria* PorB share similar properties, with both protein species being capable of binding nucleotides and exhibiting voltage-dependent gating 50. Por protein also modulates phagosome maturation by changing the phagosomal protein composition through the increase of early endocytic markers and the decrease of late endocytic markers, which ultimately delays phagosome maturation 51.

### Opacity-associated protein (Opa)

Opa proteins are integral outer membrane proteins and cause colonies to appear opaque due to inter-gonococcal aggregation when viewed by phase-contrast microscopy 52,53,54. Opa proteins belong to a multigene family with a single gonococcal cell possessing up to 12 *opa* genes that are constitutively transcribed 55,56. Each gene contains conserved, semivariable and 2 hypervariable regions, with the hypervariable segments of the proteins being located on the outside of the outer membrane 55. Opa protein expression can undergo phase variation due to changing the numbers of pentameric repeat units (-CTCTT-) that are located within the leader peptide encoding region, which results in on/off switching of expression 57. A single cell is capable of expressing either none to several different Opa proteins 57,58.

Unlike pili, Opa expression is not required for the initial attachment of gonococci to the host. However, as an infection proceeds, Opa expression varies 58, and Opa-expressing bacteria can be observed in epithelial cells and neutrophils upon re-isolation from infected human volunteers 59,60. The invasive capacity of *N. gonorrhoeae* is determined by the differential expression of Opa 61. Individual Opa proteins bind to a variety of receptors on human cells through their exposed hypervariable regions. The binding specificity for human receptors falls into two groups: OpaHS which recognize heparin sulfate proteoglycans 62,63; and, OpaCEA which recognize the carcinoembryonic antigen cell adhesion molecule (CEACAM) family that is comprised of the various CD66 molecules 64,65,66,67. CEACAMs are the major receptors of Opa proteins and are expressed on many different cell types including epithelial, neutrophil, lymphocyte and endothelial cells 68.

### Lipooligosaccharide (LOS)

As with all Gram-negative bacteria, gonococci possess lipopolysaccharide in the outer membrane. Gonococcal LPS is composed of lipid A and core polysaccharide yet lacks the repeating O-antigens 1. Accordingly, gonococcal LPS has been designated as lipooligosaccharide (LOS). Due to its surface exposure, gonococcal LOS is a primary immune target along with the major outer membrane protein Por 69,70,71. Gonococcal LOS is also toxic to fallopian tube mucosa causing the sloughing off of the ciliatory cells 72. The LOS oligosaccharide composition is highly variable both in length and in carbohydrate content. Consequently, heterogeneous LOS molecules can be produced by a single cell. However, distinct forms of LOS may be a prerequisite for infection in men 73. The most common carbohydrates associated with isolated LOS molecules are lacto-N-neotetraose (Galβ(1-4)GlcNAcβ(1-3)Galβ(1-4)Glc) and digalactoside Galα(1-4)Gal and switching from one form to another occurs at high frequency 74 through phase variation of glycosyl transferases 75,76. The variable oligosaccharide portions of LOS can also mimic host glycosphingolipids, thus promoting bacterial entry 74. In addition, gonococcal LOS can also be sialylated which renders the bacteria resistant to serum killing 77,78,79,80. Consequently, gonococcal LOS contributes to gonococcal pathogenicity by facilitating bacterial translocation across the mucosal barrier as well as by providing resistance against normal human serum 81,82.

### IgA protease

Immunoglobin A (IgA) protease is another virulence factor in *N. gonorrhoeae* 83. Upon release from the cell, the protein undergoes several endo-proteolytic cleavages, leading to maturation of the IgA protease 84. During an infection, the mature protease specifically targets and cleaves IgA1 within the proline-rich hinge region of the IgA1 heavy chain. The human IgA2 subclass is not cleaved by gonococcal IgA protease since it lacks a susceptible duplicated octameric amino acid sequence 85. *Neisseria* IgA protease also cleaves LAMP1 (a major lysosome associated membrane protein), which leads to lysosome modification and subsequent bacterial survival 86. Furthermore, *iga* mutants are defective in transcytosis of bacteria across an epithelial monolayer 87.

## PATHOGENESIS

**Figure 1:**
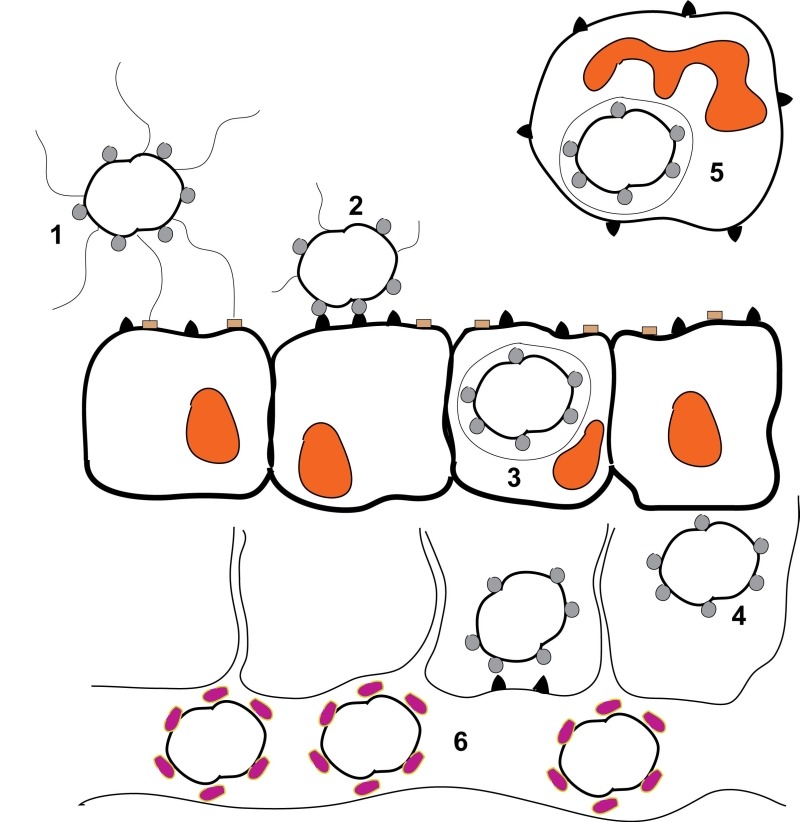
FIGURE 1: Schematic representation of a *Neisseria gonorrhoeae* infection. **1)** Piliated, Opa-expressing gonococci interact with the mucosal epithelium. The thin, hair-like pilus appendages provide the initial contact with receptors on the surface of the mucosal cells. **2)** Pili are then retracted which allows for more intimate, Opa-mediated attachment of the bacteria with the CD66 antigens located on the mucosal cells. **3)** Following Opa-mediated attachment, the bacteria are engulfed and are internalized into the mucosal cells. **4)** Following internalization, some bacteria can transcytose to the basolateral side of the mucosal epithelium. **5)** Depending upon which Opa protein is being expressed, gonococci can also reside and survive inside of neutrophils. **6)** Following transcytosis, gonococci can enter the bloodstream where heavy sialylation of lipooligosaccharide renders the bacteria serum resistant. This figure is based on 98.

*Neisseria gonorrhoeae* primarily colonizes the urogenital tract after sexual contact with an infected individual 88. The gonococcus can exist as both an extracellular and intracellular organism, with the bulk of its genes being devoted to colonization and survival, due to the fact that it cannot survive outside of a human host 89. Transmission is generally a consequence of sexual intercourse. Upon arrival into a new host, micro-colony formation commences on non-ciliated columnar epithelial cells approximately 1 to 2 hours post-infection 90,91. Once the micro-colonies achieve a cell density of approximately 100+ diplococci, cytoskeletal rearrangement and host protein aggregation occurs, which leads to pilus-mediated attachment of the gonococcus to the CD46 host cell-surface receptor (Fig. 1) 89,92. Once bound, the pilus structures on some organisms are retracted through PilE depolymerization 39 which promotes tighter contact with the host cells through Opa binding to the CEACAM receptors (Fig. 1) 65,66. Upon CEACAM binding, actin polymerization and rearrangement is induced within the host cell which results in bacterial engulfment, transcellular transcytosis and release of the bacteria into the subepithelial layer (Fig. 1) 68,93.

* In vivo*, the coordinated expression of pili and Opa varies considerably 94. Organisms isolated from the male urethra generally co-express pili and one of several Opa proteins 58. However, in women, Opa expression varies depending upon the stage of the menstrual cycle and whether or not the patient is taking oral contraceptives 94. At mid-cycle, bacteria isolated from the cervix express Opa, whereas those isolated during menses tend to be Opa negative 17. Moreover, organisms isolated from infected fallopian tubes are almost universally Opa negative, even though Opa expressing organism can be isolated from the cervix of the same patient 17. These observations can perhaps be explained by the fact that cervical secretions during menstruation contain more proteolytic enzymes than during the follicular phase. Consequently, non-Opa expressing cells may be selected due to the extreme sensitivity of Opa proteins to trypsin-like enzymes. However, with the recent studies demonstrating Opa interactions with CECAM receptors, it has been observed that fallopian epithelial tube cell cultures do not appear to express CECAM receptors 95. Nonetheless, in the absence of these receptors, gonococci were found to still adhere and invade. Consequently, CECAM expression, or the lack of it, possibly allows for *in vivo* phenotypic selection of distinct gonococcal populations on various tissues 96. Overall, Opa expression does appear to increase gonococcal fitness within the female genital tract 97. Generally, Opa expression is absent in most re-isolates from female disseminated infections.

### Inflammation 

The hallmark symptom of a non-complicated gonorrhea infection is a massive recruitment of neutrophils to the site of infection leading to the formation of a pustular discharge. Initially, Opa protein expression was suspected to be intimately involved in PMN stimulation 20,99,100,101. Subsequently, it was shown that following attachment of gonococci to the mucosa, the pro-inflammatory cytokines IL-6 and TNF-alpha as well as the chemokine IL-8 are released leading to the recruitment of neutrophils 102. In addition, upon arrival at the sub-epithelial layer, gonococci release LOS and lipoproteins which further stimulate cytokine production 103 as these outer membrane components are detected by Toll-like receptors (TLRs) on immune cells 104. Host cells also respond to bacterial peptidoglycan fragments within outer membrane vesicles via cytoplasmic NOD-like receptors (NLRs) which also contribute to the secretion of additional pro-inflammatory cytokines 105.

Despite the active recruitment of PMNs to a site of infection, gonococci can survive the oxidative and non-oxidative defense mechanisms (Fig. 1) 106. Survival appears to correlate with gonococci selectively triggering Th17-dependent host defense mechanisms by modulating expression of IL-17 107. Gonococci also must combat considerable oxidative stress by elaborating a number of different enzymes during the inflammatory response in order to detoxify superoxide anions (O2•−), hydrogen peroxide (H_2_O_2_), and hydroxyl radicals (HO•) 108,109. Gonococci must remove H_2_O_2_ because in the presence of ferrous ions the Fenton reaction is initiated (Fe^2+^ + H_2_O_2_ → Fe^3+^ + OH^.^ + OH^−^) which yields additional hydroxyl radicals 110,111. Catalase is used by the gonococcus to eliminate H_2_O_2_ (which significantly increases the organism’s ability to resist *in vitro* neutrophil killing) 112 in conjunction with a periplasmic cytochrome c peroxidase (Ccp) 110. Normally, superoxide ions are removed by superoxide dismutase enzymes (SOD) which convert superoxide to H_2_O_2_ and water. However, the majority of *N. gonorrhoeae* strains have no measurable SOD activity 108,109,110,111, suggesting that oxidants may be removed via an alternative mechanism. It appears that *N. gonorrhoeae* utilize manganese ions (Mn^2+^) to combat reactive oxygen species accumulation. Manganese accumulates within the cell through the Mn uptake system (MntABC), with Mn(II) and Mn(III) both scavenging superoxide and hydrogen peroxide molecules non-enzymatically. Furthermore, Mn(II)-pyrophosphate and Mn(III)-polyphosphate complexes are also effective in eliminating hydroxyl radicals that are formed via the Fenton reaction 110.

### The need for iron

Despite the problems associated with the Fenton reaction, iron is a vital nutrient, with pathogens expending considerable resources on scavenging the element from their human host. This becomes even more complicated during an infection, as the host responds to inflammation by limiting iron availability, as well as by decreasing free iron within the bloodstream 113. Even though humans keep their iron sequestered in iron-protein complexes such as transferrin, lactoferrin, haemoglobin, and ferritin, the *Neisseria* are capable of scavenging iron from both transferrin and haemoglobin 114, and express receptors for both transferrin and lactoferrin that provide a selective advantage within the host 115. Because *Neisseria* do not produce siderophores, they must directly extract iron from transferrin. To achieve this, the iron transport system consists of two large surface proteins, transferrin binding protein A (TbpA) and transferrin binding protein B (TbpB), with both of these proteins being found in all clinical isolates of pathogenic *Neisseria* 116. TbpA is an outer membrane transporter essential for iron uptake that binds both apo- and iron-containing transferrin with similar affinities, whereas TbpB, a surface-exposed lipoprotein, only associates with iron-bound transferrin 117. As the affinity of the bacterial receptor for iron is similar to transferrin’s affinity, this enables the gonococcus to compete with the host for this necessary nutrient 118. Subsequently, it was shown that the expression of the transferrin receptor was absolutely required for gonococcal infectivity 119.

### Serum resistance 

Bactericidal antibody-mediated killing was found to vary greatly between patients presenting genital infections 120. Subsequently, it was soon recognized that gonococcal surface components were the primary targets of antibody-dependent complement killing, with LPS-specific antibodies being the most effective at inducing bactericidal responses 121. Two forms of serum resistance were initially described; stable and unstable serum resistance 77,122. Unstable serum resistance is due to the modification of gonococcal LOS through the addition of sialic acid molecules to terminal galactose residues using cytidine 5’-monophosphate N-acetylneuraminic acid (CMP-NANA) which is abundant in human serum, as well as in various mucosal secretions and within professional phagocytes. Sialic acid transfer uses the conserved outer membrane-located enzyme 2,3-sialyltransferase 79. Sialylation of LOS mediates both the entry of gonococci into host mucosal cells as well as influencing bacterial resistance to killing by complement 82. Gonococcal cells harboring lightly sialylated LOS molecules are able to invade host epithelial cells more efficiently than heavily sialylated-LOS variants. However, lightly sialylated-LOS expressing cells are more susceptible to complement-mediated killing, whereas, heavy sialylation of LOS renders the bacteria resistant to normal human serum by masking the target sites for bactericidal antibodies 78,80 which prevents the functional activation of the complement cascade (Fig. 1) 81.

In contrast, stable serum resistance appears to be caused through the faulty insertion of the C5b-C9 membrane attack complex in serum resistant strains 123,124,125. Accompanying this defect in deposition, blocking antibody is also thought to cause the C3 complement component to be loaded onto a different site on the outer membrane such that it again hinders bactericidal killing 126. Clearly, complement resistance is important for organisms causing a disseminated infection, but its value is less clear for those organisms causing a mucosal infection. However, seminal plasma does contain an inhibitor of complement activation suggesting that there is some complement activity at the mucosa 127.

As indicated previously, the major outer membrane protein, Por, exists in two forms, Por1A and Por1B, with Por1A-expressing gonococci being most often associated with disseminated infections 42,43. Por1A-expressing gonococci also bind complement factor H more efficiently, and, as factor H down-regulates alternative complement activation, such binding helps explain serum resistance in these disseminated strains 128. Furthermore, it also helps explain species-specific complement evasion 129. Por protein also influences activation of the classical complement pathway, as Por binds to the C4b-binding protein, which again down-regulates complement activation 130. Consequently, as factor H and C4b-binding sites on the Por proteins impede functional complement deposition these may need to be modified in vaccine preparations as this may help alleviate problems associated with serum resistance 131.

### Active immunity

It has long been known that gonorrhea does not elicit a protective immune response and nor does it impart immune memory. Consequently, individuals can become repeatedly infected. Nonetheless, specific antibodies are generated within the genital tract that inhibit adherence to the mucosal epithelium, yet their persistence appears to be short-lived 18,132. Overall, the immune response to an uncomplicated genital infection remains modest 133.

The general unresponsiveness to an infection appears to stem from the organism being able to manipulate the host cell response. Transient decreases in T-cell populations occur within the bloodstream and appear to reflect Opa protein interactions with CD4^+^ T-cells which suppresses T-cell activation 134. Moreover, in contrast to Opa-mediated interactions with CEACAM antigens on other cell types, Opa-CEACAM1 T-cell interactions do not appear to cause the internalization of bacteria into the T-cells. This then leads to a dynamic re-cycling response with the T-cells that ultimately suppresses an immune response 135. Likewise, Opa-CEACAM1 interactions on B lymphocytes also inhibit antibody production 133,136. Even with dendritic cells, Opa-CEACAM1 interactions do not stimulate internalization 136. Instead, engulfment by dendritic cells is mediated through LOS interaction with DC-SIGN antigens. Consequently, as LOS molecules vary in composition, this allows the gonococcus a further opportunity for immune evasion 137. LOS molecules often activate immune cells through interaction with Toll-like receptors. However, LOS deacylation can moderate an immune response following interaction with its cognate Toll-like receptor leading to B-cell proliferation where antibody production is down-regulated 138.

Recently, an artificial estradiol-induced mouse infection model has been developed for gonococcal infections that allows for *in vivo* assessment 139. However, major differences exist between the human and mouse female genital tract. For example, the pH of the mouse vagina is higher, there is no comparable menstral cycle, fewer anaerobic commensal bacteria are present, and as the mice need to be treated with antibiotics, this aspect dramatically changes the resident flora 140. Nonetheless, the mouse infection model has yielded several interesting observations. Using the model, gonococci have been shown to moderate the murine innate immune response by stimulating IL-17 release from TH17 cells which subsequently effects other cells 107. In conjunction with transforming growth factor beta (TGF-beta), this coupled cytokine presence suppresses Th1/Th2 adaptive responses 141. Therefore, as the genital tract is rich in TGF-beta, gonococci naturally inhabit an immunosuppressive environment 142. Again, LOS and Opa expression play a major role in these responses, as LOS drives the Th17 response with Opa negatively impacting the Th1/Th2 responses 142. Further manipulation of the host response is also seen with gonococcal activation of IgM-specific memory B-cells in a T-independent manner. Consequently, this elicits a non-specific polyclonal immunoglobulin response without generating specific immunologic memory to the gonorrhea infection 143. Recently, human CEACAM transgenic mouse models have been developed for studying gonococcal *in vivo* infections 144,145. With these more refined models, gonococci were shown to readily infect and cause inflammation in the transgenic animals and that Opa-CEACAM interactions dramatically reduced exfoliation of the murine mucosal surface. As gonococci bind to human CR3 (hCR3) integrin to invade cervical cells and that human factor H bridges the interaction between the bacteria and hCR3, then future transgenic mouse models, expressing both hCR3 and human factor H, may further mimic a bona fide gonococcal infection *in vivo*.

### Antigenic variation

*Neisseria gonorrhoeae* can survive either as an extracellular organism, or, alternatively, as an intracellular organism within a variety of different cell types. Which state the organism enters depends largely on which surface components are expressed and whether these components are chemically modified or not. *N. gonorrhoeae* can modulate expression, or, the chemical character of its surface components either by phase variation, or, by antigenic variation 25. Generally, phase variation is a consequence of frame-shifting within a gene which leads to random switching between on/off states, whereas antigenic variation leads to changes in the chemical composition of some structural component. Therefore, each gonococcal cell can differentially express distinct surface antigens, in various chemical forms, which hinders recognition by host antibodies, facilitates multiple lifestyles 25 and helps explain the lack of efficacious vaccines to protect against a gonorrheal infection 21.

From genome analysis, 72 putative genes were identified that have the capacity to undergo phase variation 146. Consequently, the stochastic expression of various surface components leads to the emergence of micro-populations that allows colonization within unique environmental niches 147. Pilus expression can undergo on/off switching due to frameshifting either within the *pilE* gene 35, or, within the *pilC* gene 27. Similarly, LOS variation depends upon frameshifting within various glycosyl transferase genes which leads to the random acquisition of various sugar moieties on a varying LOS molecule 75,76. Opa expression relies exclusively on phase variation, as a series of pentameric repeats (-CTCTT-) reside towards the 5' end of each opa gene 57. Consequently, the addition or subtraction of a repeat(s) will bring each individual opa gene either in or out of frame. As expression of individual Opa proteins influence the cellular tropism of the organism with regards to internalization into either mucosal or lymphocytic cells, opa phase variation allows variable gonococcal populations to be established that have the potential to internalize into whatever cell becomes available 56,61. Consequently, phase variation confers a degree of fitness on the organism for a specific environment, yet provides little with respect to bona fide immune evasion.

Antigenic variation on the other hand confers remarkable immune evasion. Antigenic variation occurs extensively within the *pil* system as well as in some other minor systems (*maf* and *fha*) 26. Gonococci possess multiple variable *pil* genes; some are deemed silent (*pilS*) and serve as storage loci for variable pil sequence, and act in conjunction with a single expression locus, *pilE*, which encodes the PilE polypeptide. Recombination frequently occurs between *pilE* and an individual *pilS* leading to changes in the chemical composition of PilE. It is estimated that PilE can assume 108 chemical forms 148 which helps thwart an efficacious immune response due to its prominent surface location. Therefore, despite the fact that anti-pilus antibodies can be detected within the genital tract such antibodies do not recognize heterologous strains thus allowing for reinfection of an individual 18.

It is in the coordinated variation of these various surface components that allow gonococci to develop adaptive strategies where the organism can exist either externally or internally during an infection (Fig. 1). When gonococci reside externally, the organisms are generally piliated, with PilE undergoing antigenic variation which negates the various antibody clearing strategies. When coupled with the appropriate LOS composition, these organisms can also become heavily sialylated, which impedes serum killing, thus facilitating extra-cellular growth. In contrast, internalization into host cells requires the retraction of pili causing the cells to become non-piliated. When coupled with phase variation of Opa expression and a non-sialylatable LOS phenotype, the gonococcus can translocate across the mucosal epithelium at an initial stage of the infection and ultimately reside internally within various cell types 25. Eventually, infected host cells will undergo apoptosis, releasing bacteria back onto the mucosal lining, where in the presence of seminal plasma the appropriate cell surface reappears to facilitate transit into a new host 149.

### Vaccine development

Vaccine development for sexually transmitted diseases has long been a goal of the scientific community 150,151. However, given the extensive antigenic variation displayed by *N. gonorrhoeae*, coupled with suppression and manipulation of the host immune response, progress has been severely impeded. Nonetheless, in the mouse infection model, if Th1 responses can be induced, an infection will clear and immune memory can be established 152. Consequently, incorporating Th1-inducing adjuvants within any vaccine preparation may be crucial for success in this endeavor.

Two outer membrane proteins have come under considerable scrutiny as potential vaccine components; pilus constituents and the major outer membrane protein, Por. Because anti-pili antibodies were detected in vaginal secretions following an infection 18, this led to the early development of a parenteral pilus vaccine. Unfortunately, administration of this vaccine afforded partial protection only to homologous strains. Moreover, it also showed poor immunogenicity and did not stimulate an adequate antibody response at the site of infection 21,153,154. Consequently, other antigens were explored as potential vaccine candidates. As neisserial Por proteins can serve as adjuvants to B-cells, as well as stimulate Por-specific circulating Th2-cells that appear to migrate to mucosal surfaces, Por has come under considerable scrutiny 155,156. Por is also capable of stimulating dendritic cells where activation depends on Toll-like receptor 2. Therefore, as Por composition is relatively stable, this protein has become a promising vaccine candidate, especially if Th1-inducing adjuvants and Toll-like 2-inducing adjuvants can be included within any "designer" vaccine preparation 157,158,159.

However, a problem exists in the development of any vaccine in that antibodies within normal human serum bind to the gonococcal outer membrane protein Rmp with binding apparently, having important consequences with regard to serum resistance for the organism 160,161. The presence of cross-reactive Rmp antibodies also facilitates transmission 161 and women with Rmp antibody titers appear at an increased risk for infection 162. As the Rmp protein is in close association with Por protein 163 it would appear to be imperative that Rmp protein is excluded from any Por-based vaccine preparation. Nonetheless, a quiet optimism now pervades the field that an anti-gonococcal vaccine may be around the corner 152.

## MOLECULAR EPIDEMIOLOGY - A HISTORICAL REVIEW

### Auxotyping and serotyping - 70’s through the early 80’s

As public health decisions regarding transmissible pathogenic diseases rely heavily on epidemiological surveillance, it became necessary to accurately identify and characterize the different circulating strains of *N. gonorrhoeae* 164. Initially, isolates were typed through growth responses on chemically defined media 165,166 or by serotyping using common protein antigens or lipopolysaccharide 41,42,43,167. Consequently, the identification of different auxotypes allowed different *N. gonorrhoeae* strains to be typed with respect to disease severity 168,169. Subsequent Por-based serotyping allowed isolates to initially be grouped into two structurally related forms 41,44,170, which was then further refined using enzyme-linked immunosorbant assays to eventually define nine different Por-based serotypes 171.

Attempts were then made to differentiate isolates that caused uncomplicated, localized infections and those that caused disseminated gonococcal infections (DGI) 172. DGI phenotypes included an increased sensitivity to penicillin 173, unique nutritional requirements 168 coupled with serum resistance which led to increased virulence of DGI isolates 172. Subsequently, it has been shown that the majority of DGI isolates belonged to two distinct serotypes 43,174.

The emergence of antibiotic-resistant strains of *N. gonorrhoeae* identified a need to determine modes of antibiotic resistance among strains in order to monitor the development of new resistance genes, the lateral transfer of resistance genes, or the spread of resistance strains among the population. Early genetic mapping identified several genes involved in antibiotic resistance 175. Through epidemiologic studies and characterization of penicillinase-producing *N. gonorrhoeae* (PPNG), it was determined that two indeendent strains of PPNG arose in geographically separate populations; both carried the resistance gene on distinct plasmids, with one strain (linked to the Far East) being more prevalent than the strain linked to West Africa 176. Analysis of PPNG strains demonstrated that their introduction into the United States was due to returning military personnel from the Far East. Travel also contributed to global spread of these strains, as patients would encounter penicillin-resistant β-lactamase-producing *N. gonorrhoeae* following rendezvous with overseas prostitutes, which would in turn often transmit them to local prostitutes, thereby continuing their spread 169,177.

Such analysis of clinical isolates indicated that distinct reservoirs of infection could be detected based upon sexual preference. Studies revealed that homosexual men had a lower incidence of asymptomatic urethral infections and DGIs, yet more frequently acquired infections by strains that were more resistant to penicillin G, which at the time, accounted for the high failure rate of this antibiotic for rectal infections 178. Also, reservoirs for certain PPNG outbreaks could be traced back to female prostitutes, as these strains were largely absent from the homosexual community. Further epidemiological studies were able to identify gonococci that were exclusively present in both heterosexual men and women, or within homosexual male communities, thus defining sources of infection between male and female partners, prostitution and/or same sex partners 169.

### "Core group" hypothesis - late 70’s through the 80’s

As previous gonorrheal infections provide little to no immunity to subsequent infections, an alternative model for gonorrhea transmission was proposed, t suggesting that all cases of the disease are caused by a core group of individuals 179. This "core group" hypothesis, was later reinforced by the emergence and spread of PPNG from the Far East 169,179 and through clinical investigations in the United States 180,181. The persistence of isolates within a community was proposed to be due to a number of factors including the tendency for these strains to cause asymptomatic infections, or, alternatively, to have long incubation times prior to the onset of symptoms, which provided support to the theory that a core group of transmitters, most likely prostitutes, transmit the disease to many sexual partners 169. Epidemiological studies revealed that a substantial group of individuals (33%) admitted to continual sexual engagement even with the knowledge of potential exposure, or, worse, even after the onset of symptoms, and that men with new or multiple sex partners were more likely to contract gonorrhea 182,183. Consequently, five sociological trends were identified that assisted the rise of gonorrhea infections: 1) frequent changes in sex partners, 2) increased population mobility, 3) increasing gonococcal resistance to antibiotics, 4) decreased condom, diaphragm and spermicide use, and 5) increasing the use of oral contraceptives 184.

### Linkage disequilibrium - 1993

With the widespread use of serological typing, coupled with the desire for vaccine development, the classification and characterization of gonococcal strains invariably focused on investigating surface exposed antigens 185. However, the combination of auxotyping and serotyping proved to be unreliable, as these techniques did not always provide adequate resolution 186. As most pathogens are clonal with a disposition towards linkage disequilibrium, this property generally allows for classification based upon nucleotides that are present at variable sites, which in turn allows the serology, pathogenicity, host specificity and the presence of virulence genes to be mapped 185,187. However, panmictic microorganisms, such as the gonococcus, that undergo mutation and frequent recombinational exchanges, do not allow stable clones to emerge due to the randomization of alleles within a population. Consequently, this complicates epidemiological characterization. Also, as surface-exposed antigens that are used for serotyping also tend to evolve rapidly due to strong diversifying selection placed on them by the host immune system, this further compounds the problem 185. Given the above problems, it became necessary to index genes that only undergo neutral variation in order to investigate population structure, which led to analysis being focused on housekeeping genes involved in central metabolism 188. Consequently, novel methods of molecular typing were then devised to define outbreaks based on either local or global epidemiology 164.

### Multilocus enzyme electrophoresis (MLEE) - 90’s

The advent of multilocus enzyme electrophoresis (MLEE) allowed for the presence or absence of linkage disequilibrium within a population to be monitored via deviations between multiple chromosomal alleles 188,189,190,191. Indeed, this approach allowed for global epidemiological studies and permitted the identification of strains with an increased tendency to cause disease 164. Statistical analysis performed on the electrophoretic types of 227 global *N. gonorrhoeae* isolates provided evidence of a panmictic population structure, as no single pair of alleles was statistically significant for linkage disequilibrium. Additionally, it was determined that the genetic variability of isolates obtained from the same geographic location was as great as that found between all geographic locations that were analyzed. Consequently, it was concluded that the propensity for individual hosts to carry more than one genotype of *N. gonorrhoeae*, combined with natural competence for DNA transformation, promoted the highly panmictic nature of this pathogen 189.

### Multilocus sequence typing (MLST) - 90’s

However, MLEE had limitations as it could only detect a small proportion of mutations through differences in electrophoretic mobility 164,185. Therefore, nucleotide sequencing of the core gene set was then introduced leading to multilocus sequence typing (MLST) 164. This proved to be extremely effective at detecting relationships between identical or closely related isolates by characterizing them on the basis of sequence variation 164,192. While MLST typing could be readily applied to *N. meningitidis* isolates, it was initially thought that clinical isolates of *N. gonorrhoeae* could not be used, as gonococcal housekeeping genes appeared to be homologous 164,185,193. Also, as frequent recombination occurred within the organism, it was initially believed that the genetic relatedness of distant isolates may become obscured 194. However, recombinant exchanges must accrue over long time periods for relationships to be masked, and as the field of molecular epidemiology is only concerned with very short evolutionary time scales, any correlations drawn are unlikely to be skewed by recombination 192. Therefore, MLST studies did show that *N. gonorrhoeae* isolates could be typed using the same methods applied to *N. meningitidis* 164 and *N. lactamica* 18,195. It was through comparison of MLST data among the *Neisseriae*, that it was postulated that minimal interspecies recombination actually occurs among the housekeeping genes 186.

### eBURST - 2000’s

Typically, MLST allelic profiles were placed into a matrix of pairwise differences which allows for detection of identical or closely related isolates. However, these do not provide the necessary information on the evolutionary descent of genotypic clusters, nor do they identify the founder genotype 192. Additionally, in bacterial species such as *N. gonorrhoeae* that undergo frequent recombination, any relatedness that may be implied through the use of pairwise differences is highly suspect and most likely may not be phylogenetically relevant 196. To account for these concerns, the BURST (based upon related sequence types) algorithm was designed to analyze microbial MLST data by assigning defined sequence types (STs) to lineages which allowed the identification of a putative founder genotype 197.

The program was further refined with the development of the eBURST algorithm, which differentiates large MLST datasets based on isolates with the most parsimonious descent pattern from the probable founder, and allows for the identification of clone diversification yet also provides insight into the emergence of clinically relevant isolates 192. Initially, eBURST was used for analysis of quinolone-resistant *N. gonorrhoeae* (QRNG) 198. Previous epidemiological studies of quinolone resistance strains of *N. gonorrhoeae* could not determine if distinct isolates arose due to variation of an original strain or if multiple strains were concomitantly introduced into a specific geographic location 198,199,200,201. eBURST analysis determined the total number of QRNG strains that entered a country, the divergence of loci, and the time period during which the founder strains evolved 198. With the combination of MLST and eBURST analysis, disease isolates could now be defined with regard to distribution, population structure, and evolution 202. Consequently, the origins of pathogenic strains could now be determined as well as how bacterial populations respond to antibiotics and vaccines through analysis of recent evolutionary changes 203.

### CHEMOTHERAPY

*Neisseria gonorrhoeae* is rapidly evolving and has developed resistance to all previous and current antimicrobials. The recent emergence of multidrug resistant gonococcal isolates in Japan 204, France 205, and Spain 206 has provoked major concerns in public health circles worldwide, especially as drug resistance is spreading rapidly 207. Consequently, we may be entering an era of untreatable gonorrhea. Medications such as penicillin, and later, the fluoroquinolines, have each been used to treat gonorrhea in the past, however, resistance to these antimicrobial agents quickly developed, leaving limited options for gonococcal treatment 208. Currently, third generation extended-spectrum cephalosporins (ESCs); which include ceftriaxone (injectable form) and cefixime (oral form) are being prescribed. However, resistance to ESCs has also emerged with resistant isolates having been reported in 17 different countries 209,210.

The recent emergence of the first *N. gonorrhoeae* "superbug" strain in Japan (H041, which was later assigned to MLST ST7363) has been shown to exhibit extremely high-level resistance to all ESCs, including cefixime (MIC= 8 µg/ml), and ceftriaxone (MIC= 2-4 µg/ml) as well as to almost all other available therapeutic antimicrobials 204. Since the isolation of the H041 strain, other extensive drug resistance (XDR) strains have also been isolated in Quimper, France (F89 strain) 205 as well as in Catalonia, Spain 206, and both share considerable genetic and phenotypic similarity to the Japanese H041 strain. Unfortunately, transmission of these strains is augmented by the fact that XDR strains have been isolated from commercial sex workers, homosexual men, sex tourists, long distance truck drivers, and people undergoing forced migration, suggesting that these strains have the potential to spread globally 207.

*N. gonorrhoeae* are exceptional bacteria that can rapidly evolve to promote adaptation and survival within different niches of the human host. This is facilitated by their natural competence which allows DNA uptake from the environment via transformation, as well as by engaging in bacterial conjugation. Consequently, gonococci can acquire various different types of antimicrobial resistance (AMR), which include drug inactivation, modification of drug targets, changing bacterial permeability barriers, and increasing efflux properties 208,209. The acquisition of AMR genes was initially thought to occur within commensal *Neisseria* spp. that reside in the pharynx, as pharyngeal organisms are often exposed to antimicrobials, with the fixed mutations then being transferred to gonococci that mingle with the commensal bacteria 211. *Neisseria* can also obtain AMR through spontaneous mutations, although such events are comparatively rare. Many resistance determinants originate through the accumulation of chromosomal mutations, with only two known plasmid-borne genes having been described; penicillin resistance associated with the *blaTEM* plasmid 212,213,214 and tetracycline resistance associated with the *tetM* plasmid 215. Penicillinase-producing strains of *Neisseria gonorrhoeae* were first isolated in Southeast Asia and in sub-Saharan Africa 176. However, less than one percent of gonococcal clinical isolates in the US contain the β-lactamase gene, indicating that the major mechanism of penicillin resistance appears to result from accumulation of chromosomal mutations over time 214. Interestingly, the *N. gonorrhoeae* *tetM* conjugative plasmid 216 is not only self-transmissible but is also responsible for transfer of the β-lactamase plasmids to other gonococci, other *Neisseria* spp., and *E. coli* 217,218.

Chromosomal-mediated resistance to penicillin, as well as to other ESCs, generally involves modification of the penicillin binding proteins (PBP) coupled with mutations that enhance the efflux and decrease the influx of antimicrobials. Penicillin-resistant gonococcal strains typically contain 5 to 9 point mutations in the *penA* gene which encodes PBP2, the primary lethal target of the β-lactam antimicrobials 219,220. Penicillin and ESC minimum inhibitory concentrations (MICs) can also be elevated in strains carrying *mtrR* and *penB* mutations which increase efflux and decrease influx of the antimicrobials, respectively 204,205. Surprisingly, synergy between *mtrR* and *penB* mutations appears to have very little impact on resistance to cefixime which is mainly conferred by *penA* mosaic alleles 221.

Once acquired, resistance determinants contributing to decreased susceptibility or resistance to certain antibiotics are stably carried within *Neisseria* populations even when the antibiotic is withdrawn from treatment regimens 208. The persistence of resistance determinants also suggests that these factors do not cause a negative impact on the biological fitness of the gonococcus. In fact, antibiotic resistance can be linked with enhanced fitness as demonstrated with the MtrCDE efflux system that contributes to gonococcal virulence and survival during an infection 222,223. This efflux pump can recognize and expel not only hydrophobic antibiotics such as penicillin, ESCs, macrolides, tetracycline, and ciprofloxacin 224,225,226, but also antimicrobial compounds from the innate host response such as antimicrobial peptides, bile salts, and progesterone, allowing the bacteria to survive within host cells 227.

### Future directions

Due to the lack of an efficacious vaccine, control of gonococcal infections relies on appropriate antibiotic treatment, coupled with prevention, proper diagnosis, and epidemiological surveillance. Recently, novel dual antimicrobial therapy, e.g. ceftriaxone and azithromycin 228,29 or gentamicin and azithromycin 230 combination treatment, has been evaluated for treatment of uncomplicated gonorrhea. However, the emergence of concomitant resistance to the available antimicrobials has again compromised such an approach 207,208,228,231.

Previously developed antibiotics, including gentamicin, solithromycin, and ertapenem, are also now being considered as clinical isolates show a high degree of sensitivity to these antibiotics *in vitro* 232,233. The carbapenem, ertapenem, is potentially an option for ceftriaxone -resistant *N. gonorrhoeae* as these strains display relatively low MICs when treated with this agent 234. However, regimens with ertapenem are only applicable if ertapenem and ceftriaxone do not share the same resistance mechanism such as strains carrying certain *penA*, *mtrR*, and *penB* mutations which coincided with increased carbapenem MICs 209,234. Consequently, using these antimicrobials may only provide a short-term solution for combating multidrug-resistant gonorrhea 207.

To counteract this problem, new antibiotics are being developed for anti-gonococcal therapy. The novel macrolide-family of antibiotics, such as bicyclolides modithromycin (EDP-420) and EDP-322, display high activity against azithromycin-, ESC-, and multidrug-resistant gonococcal isolates *in vitro*. However, these macrolide drugs appear to cause some cross-resistance with high-level azithromycin resistance 235. The tetracycline derivatives, glycylcycline tigecycline and fluorocycline eravacycline (TP-434), have also been shown to be effective against ceftriaxone-resistant gonococci *in vitro*, yet, concerns remain regarding their usage and effectiveness 236,237. Recently, new broad-spectrum fluoroquinolones, such as avarofloxacin (JNJ-Q2) 238, delafloxacin, sitafloxacin 239, and WQ-3810 240, have displayed high potency against multidrug-resistant gonococcal isolates *in vitro* including ciprofloxacin-resistant strains. Finally, the lipoglycopeptide dalbavancin and 2-acyl carbapenems, SM-295291 and SM-369926, are among potential antimicrobials that can be used in gonorrhea treatment to a limited extent 241.

Current research has centered on exploring novel antimicrobials or compounds designed to inhibit new targets. Among these newly developed agents are a protein inhibitor (pleuromutilin BC-3781), a boron-containing inhibitor (AN3365) 242, efflux pump inhibitors, which enhance susceptibility to antimicrobials, host innate defense components and toxic metabolites 226,243, non-cytotoxic nanomaterials 244, host defense peptides- LL-37 (multifunctional cathelicidin peptide) 245, molecules that mimic host defensins, LpxC inhibitors 246, species-specific FabI inhibitors (MUT056399) 247, and inhibitors of bacterial topoisomerases (VT12-008911 and AZD0914) both of which target alternative sites other than the fluoroquinolone-binding site 248. Importantly, all these compounds possess potent *in vitro* activity against multidrug-resistant gonococcal strains 208,249. The novel spiropyrimidinetrione antibacterial compound (AZD0914) which inhibits DNA biosynthesis 250 appears to be extremely promising, as no emerging resistance has been observed in diverse multidrug-resistant gonococcal isolates 235. Consequently, AZD0914 is being seriously considered for its potential use as future oral treatment for gonococcal infections especially as it lacks cross-resistance exhibited by other fluoroquinolone antibiotics 251.

Ideally, the future treatment for gonorrhea will rely on individually-tailored regimens as clinical isolates will hopefully be rapidly characterized by novel phenotypic AMR tests and rapid genetic point-of-care antimicrobial resistance tests. Unfortunately, no commercial molecular diagnostic kit is currently available to detect AMR determinants in gonococci, with the current genetic assays lacking sensitivity and specificity 249,252. Meanwhile, healthcare initiatives need to be immediately undertaken to postpone the further widespread dissemination of ceftriaxone-resistant *N. gonorrhoeae* strains. These measures should include conducting AMR surveillance on global, national, as well as local scales, identifying treatment failures, monitoring the susceptibility of gonococcal isolates to prescribed antibiotics, and using appropriate and effective antibiotics with optimized quality and doses in gonorrhea treatment regimens 209.
